# The Effects of Laboratory Parameters on the Prognosis of COVID-19

**DOI:** 10.5152/eurasianjmed.2022.22031

**Published:** 2022-10-01

**Authors:** Fatma Kesmez Can, Handan Alay, Ayşe Albayrak, Kemalettin Özden, Sinan Yilmaz, Nurinnisa Öztürk, Zülal Özkurt, Emine Parlak, Erdal Tekin, Abdullah Osman Koçak

**Affiliations:** 1 Department of Infectious Diseases and Clinical Microbiology, Atatürk University Faculty of Medicine, Erzurum, Turkey; 2 Department of Public Health, Atatürk University Faculty of Medicine, Erzurum, Turkey; 3 Department of Biochemistry, Atatürk University Faculty of Medicine, Erzurum, Turkey; 4 Department of Emergency Medicine, Atatürk University Faculty of Medicine, Erzurum, Turkey

**Keywords:** COVID-19, laboratory parameter, infection, inflammation

## Abstract

**Objective:**

While the coronavirus disease 2019 pandemic is an ongoing issue across the world, understanding the course of the disease is important for early diagnosis and treatment. We aimed, with this study, to determine the differences between laboratory parameters in different clinical pictures of coronavirus disease 2019.

**Materials and Methods:**

The study included 443 patients who presented to Atatürk University Medical Faculty Hospital between March 15, 2020, and June 15, 2020, and were diagnosed with coronavirus disease 2019 upon a positive Real Time Polymerase Chain Reaction (RT-PCR) result. The hospitalized patients were divided into 4 groups based on their clinical status. The roles of these markers in determining the severity of coronavirus disease 2019 were statistically evaluated.

**Results:**

A total of 443 patients with RT-PCR confirmation were included in the study. The mean age was 46.0 ± 19.1 years and 54.4% of the patients were male. According to the clinical classification, 16.3% of the cases were asymptomatic, 25.7% uncomplicated, 35.7% mild/moderate, and 22.3% severe. The first 3 most frequent symptoms were cough (21.3%), fever (17.7%), and fatigue (15.5%). Hypertension (36.1%) was the major comorbidity among the patients. During the follow-up of severe cases, 39.4% developed the need for intensive care. The overall mortality rate, on the other hand, was 4.7%. Regarding laboratory parameters, procalcitonin (PCT), serum ferritin, erythrocyte sedimentation rate, C-reactive protein, neutrophil count, D-dimer, troponin, and lactate dehydrogenase were at the highest level in the severe patient group while albumin, platelet, and lymphocyte count were found to be at the lowest level in the same group. A statistically significant difference was detected between the groups (*P *< .001).

**Conclusion:**

The increase in C-reactive protein, PCT, erythrocyte sedimentation rate, ferritin, troponin, D-dimer, lactate dehydrogenase, and neutrophil count and the decrease in albumin, platelet, and lymphocyte count are significant in the severe patient group; it has been concluded that they can be used to determine the severity of coronavirus disease 2019.

## Main Points

- It was observed that clinical conditions worsened with increasing age.
- The first 3 most common symptoms were cough, fever, and malaise.
- The most common comorbid condition was hypertension.
- The increase in the number of C-reactive protein, PCT, erythrocyte sedimentation rate, ferritin, troponin, D-dimer, lactate dehydrogenase, neutrophils can be a guide in determining the severity of coronavirus disease 2019 (COVID-19).
- A decrease in the number of albumin, platelet, lymphocytes may be a guide in determining the severity of COVID-19.

## Introduction

Following the first case reported on December 31, 2019, the World Health Organization declared the coronavirus disease 2019 (COVID-19) pandemic on March 11, 2020. Still ongoing across the world, the COVID-19 pandemic has constituted a major threat to public health.^1^ The virus has been identified as a novel single-stranded RNA betacoronavirus (SARS-COV-2), similar to the severe acute respiratory syndrome coronavirus.^2^ Typical symptoms of COVID-19 are fever, cough, shortness of breath, fatigue, myalgia, and headache, while some atypical symptoms such as loss of smell and taste have also been identified. Coronavirus disease 2019 primarily targets the lung and enters the body through angiotensin-converting enzyme 2 (ACE2) receptors. Angiotensin-converting enzyme 2 receptor in the body is usually found in the lungs, nasal and oral mucosa, and in many organs such as the skin, heart, arteries, kidneys, reproductive system, and brain. Coronavirus disease 2019 presents a clinical spectrum ranging from asymptomatic condition to critical pneumonia, acute respiratory distress syndrome (ARDS), and even death.^3^ Twenty percent of the cases require hospitalization due to severe illness. Therefore, monitoring of early prognostic indicators and early intervention in COVID-19 are the fundamental measures to reduce mortality.

Studies have shown that inflammatory responses play a critical role in the progression of COVID-19.^4^ Inflammatory responses triggered by the rapid viral replication of SARS-CoV-2 and the cellular destruction may recruit macrophages and monocytes. This may induce the release of cytokines and chemokines.^5^ These cytokines and chemokines then recruit immune cells and activate immune responses, and thus may lead to a cytokine storm.^6^ Some inflammatory markers such as IL 6, IL 1β, IL 8, IL 9, and IP10 are crucial in the follow-up period in terms of disease severity and mortality.^7^

Studies show that the increase in serum C-reactive protein (CRP), procalcitonin, and ferritin levels used in clinical follow-up in COVID-19 is a significant inflammatory condition.^8^ It is thought that there is a reciprocal interaction between the cytokine storm induced by SARS-CoV-2 and the activated coagulation system. Inflammation and coagulation steps are known to cross-effect in COVID-19 patients.^9^ Disseminated intravascular coagulation (DIC) is common among mortal COVID-19 patients. Hematological parameters, including peripheral blood counts and coagulation tests, have been prominent in estimating the severity of COVID-19 from the outset.^10^ Changes in peripheral blood cell counts in patients infected with COVID-19; white blood cells and neutrophil counts increase, while lymphocyte and platelet counts decrease. Because neutrophil-to-lymphocyte ratio (NLR) is calculated based on neutrophil and lymphocyte counts, changes in NLR values may aid in the assessment of the prognosis of COVID-19 infections.^11^ Neutrophil-to-lymphocyte ratio has been shown as a marker of inflammation and prognosis in many diseases such as inflammatory bowel disease, diabetes, thyroiditis, heart diseases, and irritable bowel disease.^12^ In a study by Price-Haywood et al.^13^ it was reported that the prevalence of comorbid conditions such as diabetes, hypertension, and chronic kidney disease varies in different ethnic populations and that the accompanying symptoms such as fever, cough, or shortness of breath at the admission of COVID-19 patients differ. They also noted that biomarkers of heart damage, liver and kidney function, and inflammation and coagulation measures were significantly elevated. The effect of these variables caused the COVID-19 positivity, hospitalization, intensive care, and mortality rates to be different in different regions.^13^

With this study, of the patients with COVID-19 who we followed in our region and divided into 4 groups according to their clinical status, we aimed to determine the effect of hematological and biochemical markers such as PCT, erythrocyte sedimentation rate (ESR), CRP, lymphocyte count, neutrophil count, D-dimer, Lactate dehydrogenase (LDH), troponin, albumin, and platelet (PLT) on prognosis.

## Materials and Methods

This study was designed prospectively. The study included 443 patients who presented to Atatürk University Medical Faculty Hospital between March 15, 2020, and June 15, 2020, and were diagnosed with COVID-19 upon a positive RT-PCR result. In line with the COVID-19 Guide prepared by the Scientific Advisory Board of the Turkish Ministry of Health, the hospitalized patients were divided into 4 groups based on their clinical status. Patients’ laboratory parameters (D-Dimer, CRP, procalcitonin, ferritin, sedimentation, LDH, troponin, albumin, lymphocyte count, neutrophil count, and thrombocyte count) and comorbidities were recorded. Groups’ parameters were statistically compared with each other to be evaluated.

1. Asymptomatic group: It consists of patients with a positive RT-PCR result but showing no COVID-19 symptoms.
2. Uncomplicated group: It consists of patients with findings such as fever, muscle/joint pains, cough, and sore throat but having no respiratory distress (breath per minute 93% in room air) and presenting normal lung x-ray and/or lung tomography.
3. Mild/moderate pneumonia group: It consists of patients with findings such as fever, muscle/joint pains, cough, and sore throat, having breath per minute <30, SpO_2_ >90% in room air, and presenting mild-moderate pneumonia findings in lung x-ray or tomography.
4. Severe pneumonia group: It consists of patients with findings such as fever, muscle/joint pains, cough, and sore throat, having breath per minute ≥30, SpO_2_ ≤90% in room air, and presenting bilateral diffused pneumonia findings in lung x-ray or tomography.

### Statistical Analysis

Statistical Package for the Social Sciences v24 program was used to analyze the study data. Data were presented as mean ± standard deviation, figure, and percentage. We controlled the suitability of the data to normal distribution using the Kolmogorov–Smirnov test, z-scores obtained by dividing the skewness and kurtosis coefficients by their standard errors, and graphing methods. We used Spearman’s correlation analysis to evaluate the associations between variables. The Kruskal–Wallis test was employed to explore the distribution of laboratory parameters in the groups, and the Mann–Whitney *U*-test with Bonferroni correction was used for post hoc analyses. Analysis results were considered significant if *P *< .05.

### Ethics Committee Approval

This study was designed and submitted to the T.R. Ministry of Health COVID 19 Scientific Research Committee and Atatürk University Medical School Clinical Research Ethics Committee for approval. Approval was granted by both committees (date: May 28, 2020, number: B.30.2.ATA.0.01.00/277).

### Informed Consent

Written informed consent was obtained from patients who participated in this study.

## Results

The mean age was 46.0 ± 19.1 years and 54.4% of the patients were male. According to the clinical classification, 16.3% of the cases were asymptomatic, 25.7% uncomplicated, 35.7% mild/moderate, and 22.3% severe. Age distribution was the lowest (37.5 ± 16.29) in the asymptomatic group and the highest (60.8 ± 16.5) in the severe group, and it was observed that the clinical conditions worsened with age (*r* = 0.477, *P *< .001). The first 3 most frequent symptoms were cough (21.3%), fever (17.7%), and fatigue (15.5%). Hypertension (36.1%) was the major comorbidity among the patients. During the follow-up of severe cases, 39.4% developed the need for intensive care. The overall mortality rate, on the other hand, was 4.7% (Table 1). A statistically significant difference was detected in relation to all laboratory parameters among the clinical classification groups (Table 2).

## Discussion

Coronavirus disease 2019 may present a prognosis ranging from a clinical picture with mild symptoms to ARDS and even death. Till now, there has not been a specific treatment developed for COVID-19.^14^ Therefore, early intervention in the disease requires monitoring the markers demonstrating the progression of the disease.

In our study, 16.3% of the cases were asymptomatic, 25.7% uncomplicated, 35.7% mild/moderate, and 22.3% severe. The existing literature, on the other hand, contains mostly information on patients with mild disease. Age distribution was the lowest (37.5 ± 16.29) in the asymptomatic group and the highest (60.8 ± 16.5) in the severe group, and it was observed that the clinical conditions worsened with age (*r* = 0.477, *P *< .001). Hypertension (36.1%) was the major comorbidity. Wang et al^15^ reported in their studies that the mean age was high in the critical patient’s group and the most frequent comorbidity was cardiovascular diseases.^15^

The first 3 most frequent symptoms were cough (21.3%), fever (17.7%), and fatigue (15.5%). Studies in the current literature have reported fever, cough, and fatigue as the most frequent symptoms of COVID-19. Moreover, mortality has been reported to be higher in the elderly and comorbid patients.^16-17^ In our study, on the other hand, the overall mortality rate was 4.7%. In lung infections, the level of D-dimer is directly associated with the coagulation process during acute and chronic lung injury. Severe lung injury in severely ill patients causes overactivation of the fibrinolytic system and increased degradation of fibrin in the alveoli and thereby may lead to higher D-dimer levels. For patients with pneumonia, increased D-dimer level may be indicative of poor prognosis along with the increased inflammatory response. It has thus been suggested that the change in D-dimer may be used to evaluate the severity of the disease.^18-19^ In COVID-19, pathological episodes such as excessive inflammation (cytokine storm, endothelial, and macrophage activation), DIC, immobilization, extensive lung injury, and secondary hypoxia may cause thromboembolic events.^20^ Coagulopathy reported in COVID-19 infection can be thought to be associated with the DIC picture presenting high D-dimer and low thrombocyte count and requiring rapid intervention. In addition, COVID-19 infection or vaccines may trigger autoimmune thrombocytopenia.^21^ Asghar et al.^22^ reported in their study that D-dimer is an effective biomarker in showing the severity and mortality of the disease in COVID-19.^22^ Similarly, in our study, D-dimer and thrombocyte count levels were significantly different between the relevant clinical groups. It was observed that the severe patient group had high D-dimer and low thrombocyte. C-reactive protein is a systemic marker of the acute-phase response in inflammation, infection, and tissue damage that may be used as an indicator of inflammation.^23^ A high serum PCT level usually suggests a bacterial infection. Critically ill patients with a long duration of illness and frequently needing respiratory support are expected to be susceptible to bacterial infection.^24^ In our study, CRP and PCT levels were significantly different between the groups and were highest in the severe patient group (*P *< .001). A meta-analysis showed that increased procalcitonin values are associated with an approximately 5 times higher risk of severe SARS-CoV-2 infection. It has been reported that serial measurements of procalcitonin may be used to evaluate the progression of the disease to a more severe form.^25^ In their study with 191 COVID-19 patients, Bastuğ et al^26^ concluded that the increase in CRP is one of the strong indicators of prognosis in the comparison of intensive care unit (ICU) and non-ICU patients.

Erythrocyte sedimentation rate is a non-specific inflammatory marker essentially reflecting changes in plasma protein types.^27^ Erythrocyte sedimentation rate level was high in the severe patient group which is consistent with the current literature. We thought that this might be due to the fact that those in the severe patient group had higher inflammation and that the patients in the advanced age group had ESR levels increasing with age.^24^

Being the receptor to which COVID-19 binds, ACE 2 is also present in the heart, as it is in many organs. Coronavirus disease 2019 hence may cause cardiac involvement and lead to myocardial cell apoptosis.^17^ In our study, the severe patient group had high troponin, and there was a statistically significant difference between the groups. Similar to other studies, we associated this situation with more severe lung involvement, higher inflammatory response, and more common myocardial hypoxia in the severe patient group.^28^

The severe patients had higher neutrophil counts and lower lymphocyte counts compared to the other groups. There was a significant difference between the groups in terms of neutrophil and lymphocyte counts. High neutrophil levels may be related to strong stress reaction, secondary bacterial infection, and glucocorticoid administration in critical patients. COVID-19 is thought to affect T lymphocytes. Lymphocytes play an active role in adaptive immunity, and lymphopenia due to CD4 lymphocyte depletion has been found to be associated with the dysregulated immune response resulting from an excessive inflammatory response.^29^ Studies suggest that in COVID-19, the decrease in the total lymphocyte count leads to the depletion of several immune cells, impairment of the body’s cellular immunity function, and more severe progression of the disease.^7-30^ In their study on COVID-19, Shang et al^31^ found that the severe patient group had high neutrophil and low lymphocyte count, similar to our study results. These values have been thought to be associated with the prognosis of the disease.

Serum ferritin is a marker of stored iron. It may increase in case of inflammation, liver disease, and malignancies.^32^ Active ferritin may be produced during inflammatory diseases. Making up the majority of immune cells in the lung parenchyma and producing cytokines, macrophages are known to be responsible for ferritin secretion. Moreover, ferritin synthesis can also be increased by many inflammatory stimuli, including cytokines such as IL-6.^33^ In their study, Deng et al.^34^ determined an association between increased ferritin level and mortality rate in patients with COVID-19. Ferritin has been demonstrated to be an independent factor predicting in-hospital mortality in patients with COVID-19 in the intensive care unit.^34^ In our study, ferritin level was high in the severe patient group, and there was a statistically significant difference between the groups. This elevation may be associated with the inflammatory response that is severe in the severe COVID-19 patient group.

Albumin has an important role in nutrition and the maintenance of plasma osmolarity. It is a protein synthesized by the liver.^35^ In a study, it was stated that low albumin levels are an indicator of the patient’s poor diet, which also reduces the body’s immune response, and nutritional deficiencies often weaken the host’s immune response to RNA virus infection.^36^ Examining 78 COVID-19 patients, Liu et al.^37^ found that the serum albumin level was significantly lower in the critical patient group compared to the clinically stable patient group. Similarly, in our study, low serum albumin levels were observed in severe COVID-19 patients, and statistical significance was determined between the groups.

Lactate dehydrogenase is an intracellular enzyme found in nearly all organs. Severe infections can lead to cytokine-mediated tissue damage and LDH release. Since lung tissue has LDH, patients with severe COVID-19 infection are expected to have greater amounts of LDH in their circulation. Coronavirus disease 2019 is a severe form of interstitial pneumonia that can often evolve into acute respiratory distress syndrome. In our study, the LDH level was high in the severe patient group, and there was a statistically significant difference between the groups. In their meta-analysis, Henry et al.^38^ reported a >6-fold increase in the probability of severe disease and a >16-fold increase in the probability of death among patients with high LDH levels. They, therefore, suggested that patients’ LDH levels should be closely monitored for disease progression.

The most important limitation of this study is that it was carried out in a single center. Therefore, the number of patients remained limited when separated by clinical groups. Another limitation is the mutations that occur in SARS-CoV-2 viruses. It is thought that the low vaccination rate at the time of the study also affected the number of severely ill patients

In conclusion, the increase in CRP, PCT, ESR, ferritin, troponin, D-dimer, LDH, and neutrophil count and the decrease in albumin, PLT, and lymphocyte count are significant in the severe patient group, and it is thought that they can help in determining the severity of COVID-19. Monitoring these biomarkers will be useful for predicting disease severity, guiding treatment, and improving patients’ clinical outcomes.

## Figures and Tables

**Table 1.** Some Demographic Characteristics, Distribution of Symptoms, and Comorbidities of Cases According to Clinical Classification

	**Asymptomatic (n = 72)**	**Uncomplicated (n = 114)**	**Mild/ModeRate (n = 158)**	**Severe (n = 99)**	***P***
**Age** (mean ± SD)	37.5 ± 16.2	35.3 ± 16.0	48.4 ± 17.2	60.8 ± 16.5	**<.001**
**Sex**					
Male [n (%)]	39 (54.2)	67 (58.8)	75 (47.5)	60 (60.6)	.140
Female [n (%)]	33 (45.8)	47 (41.2)	83 (52.5)	39 (39.4)	
Symptoms ^*^					
Cough [n (%)]	-	52 (21.2)	64 (22.8)	45 (18.2)	-
Fever [n (%)]	-	38 (15.5)	42 (14.9)	55 (22.3)	-
Fatigue [n (%)]	-	37 (15.1)	49 (17.4)	34 (13.8)	-
Dyspnea [n (%)]	-	23 (9.4)	34 (12.1)	38 (15.4)	-
Sore throat [n (%)]	-	25 (10.2)	20 (7.1)	6 (2.4)	-
Headache [n (%)]	-	21 (8.6)	15 (5.3)	12 (4.9)	-
Body pain [n (%)]	-	14 (5.7)	14 (5.0)	14 (5.7)	-
Loss of appetite [n (%)]	-	8 (3.3)	13 (4.6)	13 (5.3)	-
Stomach ache [n (%)]	-	5 (2.0)	15 (5.3)	7 (2.8)	-
Loss of taste/smell [n (%)]	-	7 (2.9)	7 (2.5)	3 (1.2)	-
Diarrhea [n (%)]	-	6 (2.4)	3 (1.1)	7 (2.8)	-
Sweating [n (%)]	-	3 (1.2)	3 (1.1)	3 (1.2)	-
Dizziness [n (%)]	-	4 (1.6)	-	6 (2.4)	-
Nausea/vomiting [n (%)]	-	2 (0.9)	2 (0.8)	4 (1.6)	-
Comorbidities ^*^					
HT [n (%)]	4 (44.5)	10 (41.6)	26 (41.3)	21 (28.8)	-
DM [n (%)]	1 (11.1)	6 (25.0)	13 (20.6)	16 (21.9)	-
CAD [n (%)]	2 (22.2)	6 (25.0)	11 (17.5)	8 (11.0)	-
COPD [n (%)]	-	1 (4.2)	10 (15.9)	10 (13.7)	-
CA [n (%)]	2 (22.2)	1 (4.2)	3 (4.7)	14 (19.2)	-
CRF [n (%)]	-	-	-	4 (5.5)	-

^*^Totals exceed the number of patients since multiple answers are considered here.

HT, hypertension; DM, diabetes mellitus; CAD, coronary artery disease; COPD, chronic obstructive pulmonary disease; CA, malignant; CRF, chronic renal failure.

**Table 2.** Relationship of Some Laboratory Parameters Between Clinical Groups

	**Asymptomatic** **(n = 72)**	**Uncomplicated** **(n = 114)**	**Mild/Moderate (n = 158)**	**Severe (n = 99)**	***P***
WBC [median (IQR)]	7240.0 (3350.0)^a^	6500.0 (3590.0)^b^	6100.0 (2100.0)^a,b^	7390.0 (6800.0)	.001
NE [median (IQR)]	4650.0 (2575.0)	4055.0 (3000.0)^a^	3550.0 (2510.0)^b^	5280.0 (6700.0)^a,b^	<.001
LY [median (IQR)]	1800.0 (1125.0)^a,d^	1600.0 (900.0)^b^	1415 (875.0)^c,d^	952.0 (665.0)^a,b,c^	<.001
PLT [median (IQR)]	239 500 (65 000)^a^	230 500 (66 000)^b^	240 000 (87 000)^c^	192 000 (116 000)^a,b.c^	<.001
Alb [median (IQR)]	3.8 (0.5)^a^	3.8 (0.4)^b,d^	3.7 (0.5)^c,d^	2.9 (1.7)^a,b,c^	<.001
LDH [median (IQR)]	249.0 (79.5)^a,b^	253.0 (105.0)^c,d^	291.5 (100.0)^a,c^	367.0 (233.0)^b,d^	<.001
D-dimer [median (IQR)]	288.0 (177.5)^a,b^	335.0 (211.0)^c,d^	548.0 (471.0)^a,c,e^	2600.0 (3011.0)^b,d,e^	<.001
Troponin [median (IQR)]	2.3 (2.3)^a^	2.0 (1.8)^b,c^	3.2 (4.3)^b,d^	9.3 (45.7)^a,c,d^	<.001
ESR [median (IQR)]	8.0 (11.5)^a^	7.0 (10.0)^b,c^	18.5 (27.0)^b,d^	56.0 (39.0)^a,c,d^	<.001
CRP [median (IQR)]	4.0 (4.7)^a^	3.1 (8.0)^b,c^	8.7 (20.9)^b,d^	101.0 (103.0)^a,c,d^	<.001
Ferritin [median (IQR)]	64.0 (85.5)^a^	76.0 (105.0)^b,c^	125.5 (241.0)^b,d^	1022.0 (740.0)^a,c,d^	<.001
PCAL [median (IQR)]	0.0 (0.0)^a^	0.0 (0.1)^b^	0.0 (0.1)^c^	1.2 (2.6)^a,b,c^	<.001

^a,b,c,d,e^Post hoc analysis results show a significant difference between groups in terms of values of the same sort.

WBC, white blood cell; NE, neutrophil; LY, lymphocyte; PLT, platelet; Alb, albumin; LDH, lactate dehydrogenase; ESR, erythrocyte sedimentation rate; CRP, C-reactive protein; PCAL, procalcitonin.
